# Strict Major Histocompatibility Complex Molecule Class-Specific Binding by Co-Receptors Enforces MHC-Restricted αβ TCR Recognition during T Lineage Subset Commitment

**DOI:** 10.3389/fimmu.2013.00383

**Published:** 2013-11-22

**Authors:** Xiao-Long Li, Mai-Kun Teng, Ellis L. Reinherz, Jia-Huai Wang

**Affiliations:** ^1^School of Life Sciences, University of Science and Technology of China, Hefei, China; ^2^College of Life Sciences, Peking University, Beijing, China; ^3^Dana-Farber Cancer Institute, Harvard Medical School, Boston, MA, USA

**Keywords:** TCR, co-receptor, lineage commitment, structure, thymocyte development

## Abstract

Since the discovery of co-receptor dependent αβTCR recognition, considerable effort has been spent on elucidating the basis of CD4 and CD8 lineage commitment in the thymus. The latter is responsible for generating mature CD4 helper and CD8αβ cytotoxic T cell subsets. Although CD4^+^ and CD8^+^ T cell recognition of peptide antigens is known to be MHC class II- and MHC class I-restricted, respectively, the mechanism of single positive (SP) thymocyte lineage commitment from bipotential double-positive (DP) progenitors is not fully elucidated. Classical models to explain thymic CD4 vs. CD8 fate determination have included a stochastic selection model or instructional models. The latter are based either on strength of signal or duration of signal impacting fate. More recently, differential co-receptor gene imprinting has been shown to be involved in expression of transcription factors impacting cytotoxic T cell development. Here, we address commitment from a structural perspective, focusing on the nature of co-receptor binding to MHC molecules. By surveying 58 MHC class II and 224 MHC class I crystal structures in the Protein Data Bank, it becomes clear that CD4 cannot bind to MHC I molecules, nor can CD8αβ or CD8αα bind to MHC II molecules. Given that the co-receptor delivers Lck to phosphorylate exposed CD3 ITAMs within a peptide/MHC (pMHC)-ligated TCR complex to initiate cell signaling, this strict co-receptor recognition fosters MHC class-restricted SP thymocyte lineage commitment at the DP stage even though both co-receptors are expressed on a single cell. In short, the binding preference of an αβTCR for a peptide complexed with an MHC molecule dictates which co-receptor subsequently binds, thereby supporting development of that subset lineage. How function within the lineage is linked further to biopotential fate determination is discussed.

## Co-Receptors: Their History and Function

Two major subsets of human T lymphocytes were distinguished in the 1980s by surface expression of CD8 and CD4 as defined by monoclonal antibodies. These were shown to represent cytotoxic and helper T lymphocytes, respectively (1–4). Analysis of T cell clones revealed that CD8^+^ T cells were MHC class I restricted whereas CD4^+^ T cells were MHC class II restricted in their TCR recognition. The involvement of CD4 and CD8 in antigen-specific T cell recognition, despite their invariant structures, suggested that CD4 and CD8 might function as co-receptors in cognate recognition (4). In such a model, the co-receptors interact with conserved segments of MHC molecules (CD8 with MHCI and CD4 with MHCII) whereas the αβTCR recognizes a specific peptide bound to a polymorphic segment of the same MHC molecule. Subsequent structural studies over the last two decades validated this hypothesis, revealing the bidentate interaction of a TCR and a co-receptor with the peptide/MHC (pMHC) in a trimolecular complex (5–10). Given that recent reviews have highlighted detailed structures of the co-receptors (11, 12), these shall not be reviewed herein. Instead, features relevant to the biologic function of co-receptors in the mature peripheral T cell and thymocyte compartments are highlighted.

The CD8 transmembrane co-receptor is encoded by two distinct genes: CD8α and CD8β. Each consists of a single Ig-like domain followed by a lengthy stalk region of 30–50 residues with multiple O-glycosylated adducts, a TM helix, and a short cytoplasmic tail [reviewed in Ref. (13)]. The CD8α but not CD8β tail binds to Lck, essential for T cell signaling. Both CD8αα homodimers and CD8αβ heterodimers are found on the surface of lymphocytes, with the CD8αβ heterodimer being the dominant isoform expressed on CTL (14). The CD8αα isoform is expressed on γδ T cells, some NK cells, and a subset of intraepithelial lymphocytes (15). By contrast to CD8, CD4 comprises four Ig-like domains in tandem with a short stalk region and TM helix, but its cytoplasmic tail also binds Lck. In fact, a zinc clasp tethers Lck to the cytoplasmic tail of both CD4 and CD8α [Ref. (16) and references therein]. The major function of the co-receptor in T cell-mediated adaptive responses is to deliver Lck into the TCR-pMHC interacting system so that exposed ITAM(s) on one or more of the CD3 tails can be phosphorylated on tyrosine residues. This phosphorylation allows Zap-70 recruitment and the remainder of downstream signaling apparatus to assemble (17).

The affinity of CD4 for pMHCII is extremely weak (200 μM or higher) (18) and that of CD8αβ for pMHCI only slightly stronger (6, 19). By contrast, ∼1 μM affinities of TCR-pMHC interactions are not uncommon (20). Thus, the half-life of TCR-pMHC is ∼1000 times longer than that of CD4-pMHC; with little binding to pMHC contributed by the co-receptor ectodomain *per se*. The bidentate interaction of TCR and CD8αβ with a single agonist pMHC has been studied by a micropipette adhesion assay (21). Kinetic analysis reveals a two stage cooperative process with the first stage representing TCR dominant binding to pMHC. The second stage binding, delayed by 1 s, is Src-tyrosine kinase-dependent (i.e., presumably Lck) resulting in the CD8αβ co-receptor binding to the TCR-engaged pMHC molecule. This time delay may relate to a required reorientation of the pMHC upon TCR ligation to foster CD8 binding and/or other intracellular molecular events. This ordered and cooperative trimeric interaction favors agonist ligands and synergistically augments the bidentate binding to pMHC in turn linked to T cell signaling. Although not formally studied as yet, it appears likely that a similar cooperativity in binding processes will be operative for CD4 and TCR.

## Thymocyte Development

αβTCR signaling is essential for adaptive immune responses as well as thymocyte differentiation (22–28). However, in early thymic development, the first major checkpoint is referred to as β-selection. There, TCRβ chain gene rearrangement occurs in the absence of TCRα chain gene rearrangements such that an expressed TCRβ chain associates with the invariant pre-Tα (pTα), forming a pre-TCR on the cell surface (28). Signaling through the pre-TCR terminates subsequent TCRβ locus rearrangements, rescues cells from apoptosis and induces massive proliferation. This signaling process enables CD4^−^CD8^−^ double-negative (DN) cells to differentiate into CD4^+^CD8^+^ double-positive (DP) cells, facilitating TCRα gene rearrangements and thus generating a large αβTCR repertoire among DP thymocytes (28). DP thymocytes undergo positive selection and negative selection following self-MHC interaction via their αβTCR. During positive selection, DP cells mature into single positive (SP) CD4^+^ or CD8^+^ thymocytes and may egress into the thymic medulla. After 2 weeks or so of maturation therein, these SP cells migrate out to the periphery (29, 30). During negative selection, strongly self-reactive thymocytes undergo cell death. Those apoptotic thymocytes are purged from the T lineage repertoire [reviewed in Ref. (31–33)].

One remarkable feature of thymic development is the presence of the numerically major subpopulation of DP thymocytes expressing both CD4 and CD8 co-receptors. This DP phenotype is absent from human and murine peripheral T lineages (34). As Lck is critical for TCR activation and physically associated with both CD4 and CD8α and DP thymocytes express both of these co-receptors, it follows that co-receptor-based pMHC interaction will impose MHC-restricted recognition on the mature αβTCR repertoire. This was recently tested in an elegant experiment with knockout mice lacking both co-receptors and MHC class I and MHC class II proteins. In such “Quattro” knockout mice, Lck is not sequestered away from the TCR so that TCR functions in the absence of co-receptor engagement. Hence, TCR signaling is mediated through non-MHC-ligands. Such αβTCRs possess antibody-like recognition specificities (35, 36).

While the CD4 ectodomain is a rigid concatemer of four Ig-like domains with no known developmentally regulated glycosylation and hence, “tuneability,” the structural features of CD8 are quite different (13). The combined presence of *O*-glycans and prolines in the CD8 subunit stalks suggests that these membrane connectors adopt extended and somewhat stiff conformations, similar to that observed for leukosialin and mucins, nonetheless with some conformational flexibility (37, 38). This CD8αβ co-receptor flexibility likely accommodates to the variability of TCRαβ docking onto pMHCI [reviewed in Ref. (11)]. In addition, developmentally regulated glycosylation of the CD8αβ stalk modulates pMHC binding (13). Immature DP thymocytes bind MHCI more avidly than CD8 SP thymocytes. This differential binding is governed by developmentally programed *O*-glycan modification of several CD8 stalk threonine residues proximal to the CD8β headpiece and controlled by ST3Gal-I sialyltransferase (13, 39). ST3Gal-I specifically localizes to the medulla of the thymus where SP thymocytes reside. ST3Gal-I induction and attendant core 1 sialic acid addition to CD8β on mature thymocytes decreases CD8αβ-MHCI avidity by altering CD8αβ domain-domain association and/or orientation. Hence, glycans on the CD8β stalk appear to modulate the ability of the distal binding surface of the dimeric CD8 globular head domains to clamp MHCI. The DP stage facilitates efficient elimination by negative selection of autoreactive TCR specificities through this enhanced co-receptor function working in tandem with highly specific TCR-pMHCI triggered apoptosis (24). Once a thymocyte has differentiated to the CD8 SP stage, however, CD8αβ O-glycan sialylation reduces the strength of the CD8αβ co-receptor interaction with pMHCI, mandating a greater requirement for TCR-pMHCI interaction to achieve a subsequent activation threshold in mature T lineage cells (13). At the DP thymocyte stage, the higher avidity CD8αβ interaction with pMHCI will further enforce MHC-restricted αβTCR recognition via Lck delivery.

## Lineage Fate

The differentiation of DP thymocytes into CD4^+^ or CD8^+^ SP thymocytes is a fundamental example of bipotential cell fate determination. How this process is controlled during positive selection events in the thymus has been the subject of intense scrutiny [reviewed in Ref. (40)]. A stoichastic selection model that postulates that co-receptor gene expression occurs randomly has been largely discredited (41). Instructional models postulating that TCR signals during positive selection specifically direct termination of irrelevant co-receptors have been controversial. One early strength of signal model was popular because the CD4 cytoplasmic tail manifest stronger affinity for Lck than that of CD8α, engendering TCR/CD4 co-engagement signals stronger than that of TCR/CD8 (42, 43). However, genetic manipulation of ITAMs within the TCR complex did not change CD4 vs. CD8 lineage choice (44). A more recent variant of the instructional model is a duration of signal model. In this view, TCR signaling of long duration terminates CD8 gene expression while TCR signaling of short duration terminates CD4 gene expression. Recent data have shown that positive selection in DP thymocytes *per se* invariably decreases surface CD8, thereby selectively disrupting MHC class I-restricted signaling (45). The kinetic signaling model proposed by Singer et al. incorporates aspects of this signaling duration model in conjunction with more recent common cytokine receptor γ-chain (γC) signaling biology (40, 46).

In the kinetic signaling model, positive selection and lineage choice are sequential events. Positive selection induces all DP thymocytes, regardless of TCR specificity, to transiently terminate CD8 gene expression resulting in CD4^+^CD8^low^ thymocytes, in part by suppressing E8_III_ *Cd8* enhancer activity (47). Persistent TCR engagement blocks IL-7 signaling and fosters CD4^+^ SP development with loss of CD8. In contrast, disruption of TCR engagement permits IL-7 signaling and results in “co-receptor reversal” with CD8 SP development [reviewed in Ref. (47)]. IL-7 signaling induces RUNX3 transcription that initiates CD8 expression via the E8_III_ *Cd8* enhancer and also activates the cytotoxic gene transcriptome in CD8 T cells. The *Cd8* gene locus *cis* elements control thymocyte lineage fate with respect to the cytotoxic vs. helper lineage program (47).

The above thymocyte biology now needs to be interpreted in a structural context as relates to co-receptor and pMHC interactions in order to understand bipotential DP thymocyte cell fate determination in the DP to SP developmental transition. Here we offer insights into this process with respect to MHC-restricted lineage development and linkage to mature T cell function.

## Conserved Geometry of MHCII α2/β2 Domains for CD4-Binding

Structures of CD4/MHCII as well as CD8/MHCI complexes have been solved. These reveal two very different binding modes. CD4 solely uses its N-terminal domain for insertion into a hydrophobic cave-like structure formed by the two membrane-proximal domains of the MHCII molecule (α2/β2). In contrast, CD8 uses its two dimeric Ig-like domains to clamp onto the CD loop of the MHCI α3 domain in a fashion similar to an antibody binding to an antigen (5–7).

**Figure 1:**
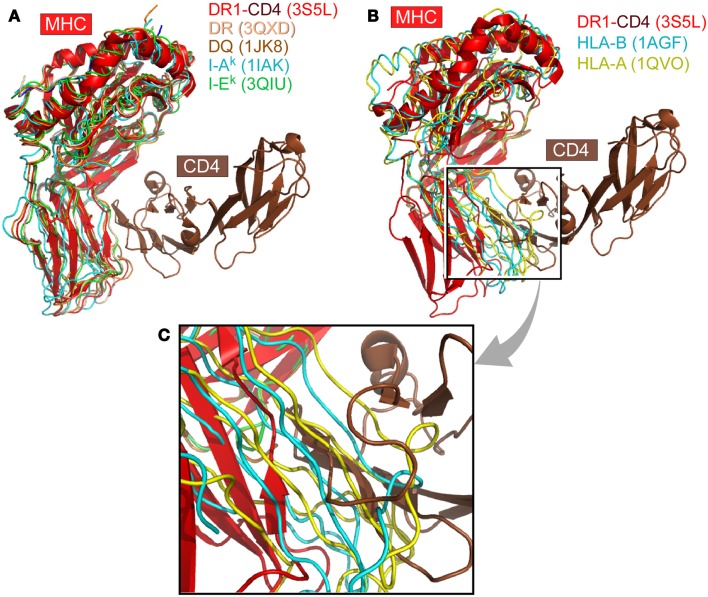
**The CD4 co-receptor binds to class II but not class I MHC molecules**. **(A)** Superposition of selected MHCII molecules onto the structure of the MHCII molecule DR1 in complex with the N-terminal two-domain of CD4 (PDB code: 3S5L). The superposition is based on the α2 domain. In all cases, the MHCII molecules can accommodate the CD4 N-terminal domain for binding. **(B)** Two MHCI molecules are superimposed onto the DR1-CD4 complex structure. Here the β2M domain of MHCI was overlaid on the α2 domain of DR1 molecule. These two selected MHCI molecules have the largest (1AGF, in blue) and smallest (1QVO, in yellow) opening angle, respectively, between the two membrane-proximal domains, α3 and β2M. Compared with the MHCII DR1-CD4 complex, they do not have a sufficiently large opening angle to accommodate CD4. **(C)** Close-up of **(B)**.

We have systematically surveyed herein 58 MHCII structures and 224 MHCI structures in the protein data bank (PDB). The structure of human CD4 in complex with HLA-DR1 (PDB code 3S5L) was taken as a reference (48). All other MHC structures were overlaid onto HLA-DR1 within this 3S5L structure. The superposition was based on the α2 domain in the case of MHCII molecules or the corresponding β2M domain in the case of MHCI molecules. Figure 1 depicts this overlay of selected MHC molecules onto HLA-DR1 in the CD4/HLA-DR1 complex. The representative MHCII molecules from human or mouse all preserve an angle between the two membrane-proximal α2/β2 domains that can readily accommodate CD4-binding (Figure 1A). On the contrary, Figures 1B,C show two selected MHCI molecules overlaid, which have largest (1AGF) or smallest (1QVO) opening angle between respective membrane-proximal β2M/α3 domains. Clearly the MHCI molecules’ geometry appears to prevent CD4-binding.

We have superimposed a representative group of MHCII molecules onto mouse I-A^k^ in the complex structure of D10 TCR/I-A^k^ (1D9K) based on the two membrane-proximal α2/β2 domains, several of which are pictured in Figure 1A. A larger set includes human HLA-DR1/TCR complex (1FYT), HLA-DRA/TCR complex (1YMM), HLA-DR2a/TCR complex (1ZGL), HLA-DQ8 (1JK8) as well as mouse I-A^u^/TCR complex (1U3H), I-E^k^ (1IEA), and CD4/I-A^k^ complex (1JL4). The RMSD value for 178 overlaid Cα atoms ranges from 0.63 to 1.1 Å, indicating that the two membrane-proximal domains as a structural unit have a conserved relative angle. The same calculation was carried out for MHCI molecules. A representative group of MHCI molecules was superimposed onto H2-K^b^ in the H2-K^b^/CD8αα complex structure (1BQH) based on the two membrane-proximal β2M/α3 domains. These include human HLA-A (1HHJ), HLA-B (1AGB), HLA-C (1IM9), HLA-A/TCR complex (1AO7), HLA-A2/CD8αα complex (1AKJ), as well as mouse H2-K^b^/TCR complex (2CKB) and mouse H2-D^b^ (1QLF). The RMSD value for about 180 Cα atoms ranges from 0.73 to 1.49 Å, indicating that the two membrane-proximal domains as a structural unit also have a relatively fixed angle. Notably, the RMSD value for MHCI is slightly larger than that of MHCII, consistent with previous observation that MHCI α3 domain has a small angular variation relative to the rest of MHCI molecule (5). We further compared the separation angles of the two membrane-proximal domains between the two classes of MHC molecules. We took the class II molecule HLA-DQ8 (1JK8) and class I molecule H2-K^b^ in its CD8 complex (1BQH) for comparison. If the MHCII β2 domain is superimposed onto the MHCI α3 domain, the rest of the MHCII molecule has about a 38° rotation relative to the counterpart of MHCI. This manifests as a larger opening between α2 and β2 domains in MHCII compared to between β2M and α3 in MHCI, a geometric requirement for CD4-binding as shown in Figure 1B. In that figure, MHCII α2 domain and MHCI β2M domain were overlaid in the back. To accommodate CD4, the red-colored front β2 domain of MHCII molecule DR1 appears to “swing” leftwards compared to its counterparts α3 domain of MHCI molecules HLA-A (in yellow) and HLA-B (in blue).

**Figure 2:**
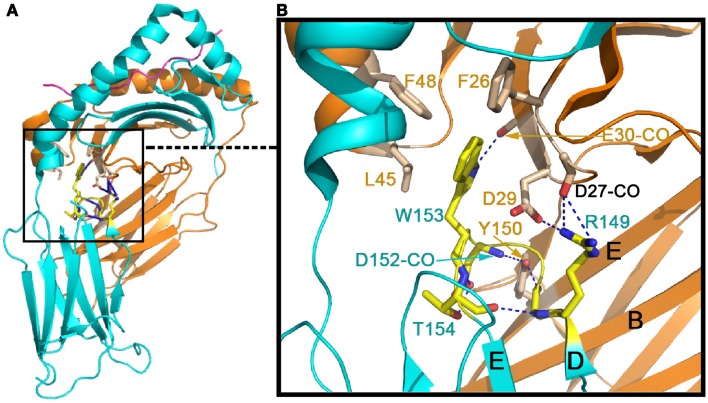
**Conserved inter-domain interface in the MHCII molecule HLA-DQ8 (1JK8) that maintains the inter-domain orientation for CD4-binding**. **(A)** The overall view of HLA-DQ8. The gold color indicates the α chain whereas the cyan color defines the β chain. **(B)** Zoomed-in view of detailed inter-domain interaction involving particularly W153 at the β chain’s DE loop (in yellow) and Y150 on the α chain’s E strand (in light brown).

Figure 2 shows the conserved domain-domain interface interaction in the MHCII molecules HLA-DQ8 (1JK8). Figure 2A is the overview of the structure, whereas the Figure 2B is a zoomed-in view of the α2/β2 domain interface revealing atomic interaction in detail. One critical residue is an invariant Trp153 of the β2 domain (Trp153β) that forms a hydrogen bond to the main chain carbonyl oxygen of Glu30α from α1 domain. In addition, the indole ring of this Trp153β is surrounded by three conserved hydrophobic residues (Phe26α, Leu45α, and Phe48α) of the α1 domain. Since Trp153β sits on the DE loop, which is itself rigidified by an internal hydrogen-bond network within the loop (shown in blue broken lines), this network may play a key role in fixing the β2 domain’s relative position. Also seen in Figure 2 is an invariant Tyr150α of the α2 domain that forms a hydrogen bond to the main chain carbonyl oxygen of Asp152β, just next to Trp153β. This Tyr150α is located at the E strand of α2 domain’s framework, and along with other interaction shown in Figure 2 constrains the α2 domain so that it maintains a relatively fixed orientation with respect to the β2 domain. These interface interactions ensure that the α2/β2 domains are positioned suitably for insertion of the N-terminal domain of CD4 during binding.

**Figure 3:**
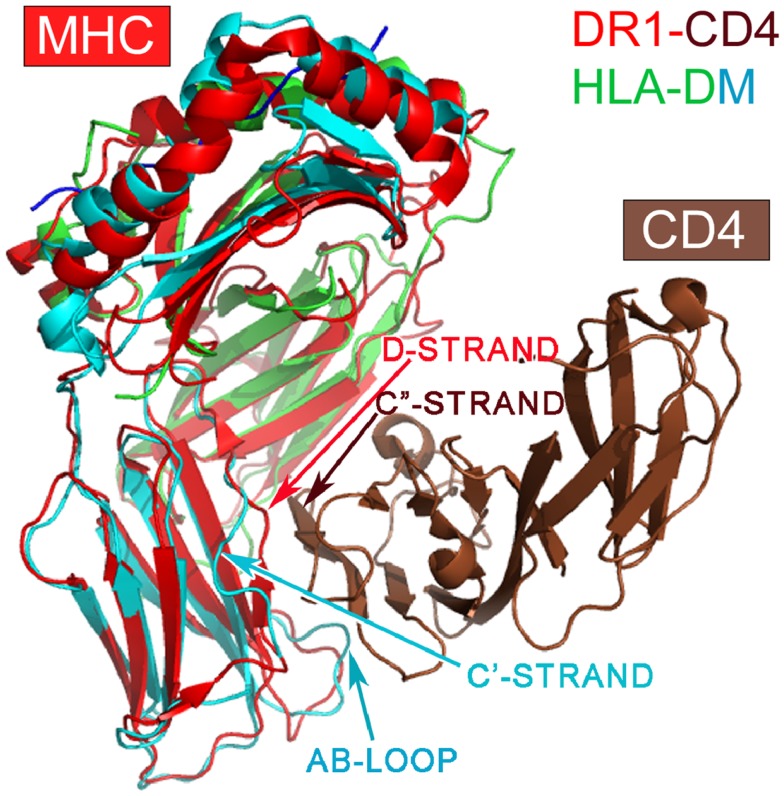
**Superposition of the HLA-DM structure onto the DR1-CD4 complex**. The superposition is based on the α2 domain. HLA-DM’s α2 domain is in green, whereas the β2 domain is in cyan. Note that DM’s β2 domain does not have a D-strand for pairing to CD4’s C″-strand necessary for docking, a key feature of CD4-binding to all MHCII molecules. In addition, DM’s AB loop protrudes, thereby colliding with CD4.

Among class II MHC-like molecules, it is interesting to note that the HLA-DM (1HDM) is structurally distinct from other class II MHC molecules (49) (Figure 3). HLA-DM has a C′ strand instead of a D-strand in its β2 domain. In all other MHCII molecules, the D strand plays a critic role in forming a mini-β sheet with the CD4 C″ strand upon binding (7). Additionally, HLA-DM has a distinct AB loop protruding out from its β2 domain that would prevent CD4-binding. DM is not expressed as a cell surface molecule, instead functioning intracellularly to facilitate peptide loading into other MHCII molecules. It would appear that HLA-DM is without any CD4-binding features, consistent with its intracellular role.

## The Invariant Gln226 of MHCI Molecules is the Determinate Residue for CD8-Binding

**Figure 4:**
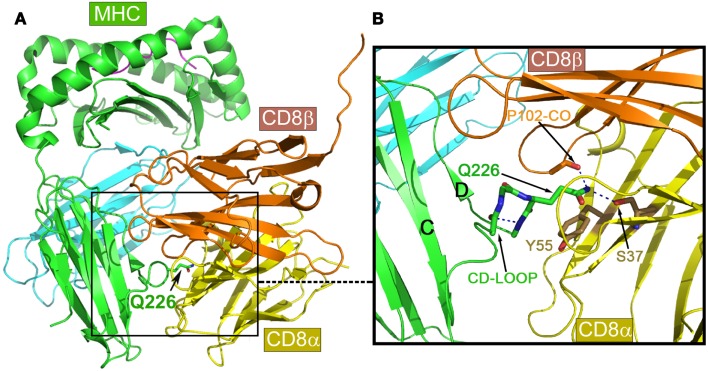
**Structure of mouse CD8αβ in complex with the MHCI molecule H-2D^d^**. **(A)** An overview of the complex. **(B)** Details of the binding region. Note that the key conserved Q226 from MHCI molecule’s CD loop pokes into a crevice formed between CD8α and β subunits, making hydrogen bonds to conserved S37 of the CD8α subunit, while the aliphatic portion of this Q226 is involved in extensive hydrophobic contacts with the conserved Y55 of the CD8α subunit.

Figure 4A is the overview of the structure of mouse CD8αβ heterodimer in complex with MHCI molecule H-2D^d^ (9). The overall structure is in a good agreement with previously published complex structures including the mouse CD8αα/H-2K^b^ (50) and human CD8αα/HLA-A2 (5) as well as mouse CD8αα homodimer in complex with non-classic MHCIb molecule TL (51). That CD8αβ/H-2D^d^ complex interaction is clearly distinct from CD4-binding to an MHCII molecule shown in Figure 1. The major CD8-binding site is the CD loop of MHCI molecule’s α3 domain, most notably containing Q226. Importantly, this α3 domain is the sole contact region for the CD8αβ co-receptor, essential for cytotoxic T cell function (9).

Of note, there is a very similar CD loop in the β2 domain of MHCII molecules (7). However, what makes MHCI molecules unique with respect to CD8-binding is the invariant Gln226 at the tip of their α3 domain CD loop. The functional importance of this Gln226 within the CD loop was first reported in 1990 (52). Later, it was further found that this Gln226, but not acidic residues on the CD loop was critically required (53). Figure 4B is a detailed view of the Gln226 contact area in the structure of the CD8αβ/H-2D^d^ (3DMM). The very tip amide nitrogen forms bifurcated hydrogen bonds to the Ser37 side chain of the CD8α subunit (in yellow) and the main chain oxygen of CD8β subunit’s Pro102 (in gold). Interestingly, we have noticed that the these hydrogen bonds pull the side chain of Gln226 into an extended conformation such that the elongated aliphatic portion of this Gln226 side chain packs onto the aromatic ring of CD8α Tyr55. This hydrophobic interaction conceivably contributes a significant amount of binding energy. Remarkably the Ser37 and Tyr55 are conserved only in CD8α, but not in CD8β subunit, which structurally positions CD8α subunit at the bottom and CD8β subunit on the top shown in the Figure 4. The MHCI CD loop was noticed to have an extensive internal hydrogen-bond network to rigidify the loop, supporting the Gln226 for binding (50). These CD8-binding structural features observed in CD8αβ/H-2D^d^ are shared in all known CD8αα/MHCI complex structures. Upon CD8 binding to MHCI, the MHCI molecules’ buried surface area for structures of HLA-A2/CD8αα, H-2K^b^/CD8αα, TL/CD8αα and H-2D^d^/CD8αβ is 1074, 1557, 1645, and 781 Å^2^, among which a single Gln226 residue alone contributes 16, 11, 10, and 23%, respectively. Interestingly for the H-2D^d^/CD8αβ complex, since only the MHCI α3 domain is involved in binding, the buried surface area is smallest but with Gln226 makes the highest contribution.

In summary, whereas MHCII molecules have their α2/β2 domains arranged in a conserved and opened conformation suitable for CD4 N-terminal domain insertion for binding, MHCI molecules are unique in having a key invariant Gln226 at the tip of their α3 domain’s CD loop. The latter is well-positioned to extend into the crevice formed between two CD8 subunits for specific interactions. These observations apply to the 58 MHCII and 224 MHCI structures of both human and murine origin examined.

## Sequential αβTCR-pMHC Interaction and Co-Receptor Delivery of Lck is Linked to CD4/8 Gene Imprinting

The above structural scrutiny leads us to propose the following heuristic. On developing DP thymocytes, there are clonally distributed MHCI- or MHCII-restricted TCRs that can potentially interact with the antigen presenting platforms of MHCI (α1α2 domains) and MHCII (α1β1 domains) molecules extending from the stromal cell surface. The interacting domains of both TCR and MHC molecules have no intrinsic structural differences that distinguish the two classes of MHC interactions; nor are TCR complex components distinct in overall structural features vis-à-vis the individual subunits (11). It is difficult, therefore, to conceive that such TCR-pMHC interactions would direct a differential switch-off of either CD4 or CD8 co-receptors. Instead, the preference of the TCR for a specific pMHC dictates which co-receptor then enters into the preformed TCR-pMHC complex. During positive selection, DP thymocytes first become intermediate cells (CD4^+^CD8^low^). If during positive selection, one MHCII-restricted TCR on the CD4^+^CD8^low^ cell surface encounters an MHCII molecule that presents a self-peptide on the stromal cell, the “snug” fit of CD4 into the MHCII molecule’s α2/β2 domains on the cell surface as described above brings Lck into close proximity with exposed CD3 cytoplasmic tail ITAMs in the TCR complex. If successful ITAM phosphorylation and downstream activation persists, transcriptional factors like GATA3 and Th-POK are induced and that induction promotes CD4^+^ T cell differentiation by preventing *Cd4* gene silencing (40). This successful signaling eventually terminates any CD8 expression, and the thymocyte differentiates into CD4^+^ SP cell. If during positive selection an MHCI-restricted TCR on one CD4^+^CD8^low^ thymocyte surface encounters an MHCI that presents a self-peptide, then CD4 fails to contact the MHCI molecule. Consequently, TCR signaling is disrupted with loss of GATA3 and Th-POK transcription. Instead, IL-7R signaling is initiated to up-regulate RUNX3, thereby silencing *Cd4* and activating *Cd8* expression. CD8 co-receptor function is operative. As RUNX3 is also linked to the cytotoxic program, this dichotomy normally ensures that CD8 SP development is coupled to the CTL program.

As both human and mouse TCRs manifest ∼10-fold greater 3-D binding affinities for pMHCI compared to pMHCII cognate ligands (54, 55), the relatively persistent interaction of class II MHC-restricted TCRs with pMHCI ligands in the thymus as postulated above may appear paradoxical. However, recent studies show that TCR-triggered interaction with pMHC is force dependent, dramatically modifying TCR-pMHC bond lifetimes (56, 57). Such bond lifetime alteration is not predicted by 3-D affinity measurements. Furthermore, the trimolecular TCR-pMHC-co-receptor interaction likely alters the landscape of bond lifetimes further, particularly under a force load, as would occur when thymocytes scan thymic epithelial cells. That said, other differences in CD4 vs. CD8 lineage-specific death rates at the DP thymocyte branch point imply additional biological parameters modulating developmental progression (58).

## Concluding Remarks

Since the introduction of TCR transgenic mouse studies, it has been clear that the interaction of the TCR in a DP thymocyte with a specific thymic pMHC molecule determines differentiation of that DP thymocyte to either a CD4 or CD8 SP thymocyte (59). The paradox that there were no overall architectural distinctions between TCRs that recognize peptides bound to MHCI vs. bound to MHCII molecules as revealed by structural analysis made this finding enigmatic [reviewed in Ref. (11)]. From our extensive structure data survey, it is now clear that the ability of CD4 to bind pMHCII and CD8 to bind to pMHCI molecules is inviolate. TCR recognition of antigen is MHC-restricted due to the requirement for co-receptor-mediated delivery of Lck to the TCR-pMHC complex during positive selection. Transient loss of CD8 expression that accompanies positive selection of all DP thymocytes supports a prolonged CD4 co-receptor dependent interaction of class II MHC-restricted TCRs on thymocytes with pMHCII on stromal cells. By comparison, the corresponding CD8 co-receptor dependent interaction of class I MHC-restricted TCRs with pMHCI is attenuated. In the latter case, TCR-pMHCI signaling is disrupted, IL-7R signaling permitted and consequently, RUNX3 triggers the cytotoxic transcriptome program. Thus, the specificity for CD4 vs. CD8 SP lineage commitment is coupled to the co-receptor based on a given TCR’s pMHC class preference. In contrast, effector function is linked to cytokine receptor-mediated signaling events that control downstream cellular transcriptional programs.

## Conflict of Interest Statement

The authors declare that the research was conducted in the absence of any commercial or financial relationships that could be construed as a potential conflict of interest.

## References

[1] Reinherz EL Schlossman SF The differentiation and function of human T lymphocytes. Cell (1980) 19:821–7 10.1016/0092-8674(80)90072-0 6991122

[2] Meuer SC Schlossman SF Reinherz EL Clonal analysis of human cytotoxic T lymphocytes: T4+ and T8+ effector T cells recognize products of different major histocompatibility complex regions. Proc Natl Acad Sci U S A (1982) 79:4395–9 10.1073/pnas.79.14.4395 6981813 PMC346678

[3] Meuer SC Fitzgerald KA Hussey RE Hodgdon JC Schlossman SF Reinherz EL Clonotypic structures involved in antigen-specific human T cell function. Relationship to the T3 molecular complex. J Exp Med (1983) 157:705–19 10.1084/jem.157.2.705 6185617 PMC2186929

[4] Reinherz EL Meuer SC Schlossman SF The delineation of antigen receptors on human T lymphocytes. Immunol Today (1983) 4:5–8 10.1016/0167-5699(83)90094-4 25290891

[5] Gao GF Tormo J Gerth UC Wyer JR McMichael AJ Stuart DI Crystal structure of the complex between human CD8alpha(alpha) and HLA-A2. Nature (1997) 387:630–4 10.1038/42523 9177355

[6] Kern P Hussey RE Spoerl R Reinherz EL Chang HC Expression, purification, and functional analysis of murine ectodomain fragments of CD8alphaalpha and CD8alphabeta dimers. J Biol Chem (1999) 274:27237–43 10.1074/jbc.274.38.27237 10480942

[7] Wang J-H Meijers R Xiong Y Liu J-H Sakihama T Zhang R Crystal structure of the human CD4 N-terminal two domain fragment complexed to a class II MHC molecule. Proc Natl Acad Sci U S A (2001) 98:10799–804 10.1073/pnas.191124098 11535811 PMC59561

[8] Chang HC Tan K Ouyang J Parisini E Liu JH Le Y Structural and mutational analyses of a CD8alphabeta heterodimer and comparison with the CD8alphaalpha homodimer. Immunity (2005) 23:661–71 10.1016/j.immuni.2005.11.002 16356863

[9] Wang R Natarajan K Margulies DH Structural basis of the CD8 alpha beta/MHC class I interaction: focused recognition orients CD8 beta to a T cell proximal position. J Immunol (2009) 183:2554–64 10.4049/jimmunol.0901276 19625641 PMC2782705

[10] Yin Y Wang XX Mariuzza RA Crystal structure of a complete ternary complex of T-cell receptor, peptide-MHC, and CD4. Proc Natl Acad Sci U S A (2012) 109:5405–10 10.1073/pnas.1118801109 22431638 PMC3325661

[11] Wang JH Reinherz EL The structural basis of αβ T lineage immune recognition: TCR docking topologies, mechanotransduction and co-receptor function. Immunol Rev (2012) 250:102–19 10.1111/j.1600-065X.2012.01161.x 23046125 PMC3694212

[12] Li Y Yin Y Mariuzza RA Structural and biophysical insights into the role of CD4 and CD8 in T cell activation. Front Immunol (2013) 4:206 10.3389/fimmu.2013.00206 23885256 PMC3717711

[13] Moody AM Chui D Reche PA Priatel JJ Marth JD Reinherz EL Developmentally regulated glycosylation of the CD8alphabeta coreceptor stalk modulates ligand binding. Cell (2001) 107:501–12 10.1016/S0092-8674(01)00577-3 11719190

[14] Moebius U Kober G Griscelli AL Hercend T Meuer SC Expression of different CD8 isoforms on distinct human lymphocyte subpopulations. Eur J Immunol (1991) 21:1793–800 10.1002/eji.1830210803 1831127

[15] Poussier P Julius M Thymus independent T cell development and selection in the intestinal epithelium. Annu Rev Immunol (1994) 12:521–53 10.1146/annurev.iy.12.040194.002513 8011290

[16] Kim PW Sun ZY Blacklow SC Wagner G Eck MJ A zinc clasp structure tethers Lck to T cell coreceptors CD4 and CD8. Science (2003) 301:1725–8 10.1126/science.1085643 14500983

[17] Weiss A T cell antigen receptor signal transduction: a tale of tails and cytoplasmic protein-tyrosine kinases. Cell (1993) 73:209–12 10.1016/0092-8674(93)90221-B 8477442

[18] Xiong Y Kern P Chang H Reinherz E T cell receptor binding to a pMHCII ligand Is kinetically distinct from and Independent of CD4. J Biol Chem (2001) 276:5659–67 10.1074/jbc.M009580200 11106664

[19] Gao GF Rao Z Bell JI Molecular coordination of alphabeta T-cell receptors and coreceptors CD8 and CD4 in their recognition of peptide-MHC ligands. Trends Immunol (2002) 23:408–13 10.1016/S1471-4906(02)02282-2 12133804

[20] Stone JD Chervin AS Kranz DM T-cell receptor binding affinities and kinetics: impact on T-cell activity and specificity. Immunology (2009) 126:165–76 10.1111/j.1365-2567.2008.03015.x 19125887 PMC2632691

[21] Jiang N Huang J Edwards LJ Liu B Zhang Y Beal CD Two-stage cooperative T cell receptor-peptide major histocompatibility complex-CD8 trimolecular interactions amplify antigen discrimination. Immunity (2011) 34:13–23 10.1016/j.immuni.2010.12.017 21256056 PMC3381515

[22] Kappler JW Roehm N Marrack P T cell tolerance by clonal elimination in the thymus. Cell (1987) 49:273–80 10.1016/0092-8674(87)90568-X 3494522

[23] Kisielow P Blüthmann H Staerz UD Steinmetz M von Boehmer H Tolerance in T-cell-receptor transgenic mice involves deletion of nonmature CD4+8+ thymocytes. Nature (1988) 333:742–6 10.1038/333742a0 3260350

[24] Ghendler Y Teng M-K Liu JH Witte T Liu J Kim KS Differential thymic selection outcomes stimulated by focal structural alteration in peptide/major histocompatibility complex ligands. Proc Natl Acad Sci U S A (1998) 95:10061–6 10.1073/pnas.95.17.10061 9707600 PMC21461

[25] Mariathasan S Jones RG Ohashi PS Signals involved in thymocyte positive and negative selection. Semin Immunol (1999) 11:263–72 10.1006/smim.1999.0182 10441212

[26] Love PE Lee J Shores EW Critical relationship between TCR signaling potential and TCR affinity during thymocyte selection. J Immunol (2000) 165:3080–7 10975819 10.4049/jimmunol.165.6.3080

[27] Haks MC Pepin E van den Brakel JHN Smeele SAA Belkowski SM Kessels HW Contribution of the T cell receptor-associated CD3γ-ITAM to thymocyte selection. J Exp Med (2002) 196:1–13 10.1084/jem.20020268 12093866 PMC2194018

[28] von Boehmer H Aifantis I Gounari F Azogui O Haughn L Apostolou I Thymic selection revisited: how essential is it? Immunol Rev (2003) 191:62–78 10.1034/j.1600-065X.2003.00010.x 12614352

[29] Starr TK Jameson SC Hogquist KA Positive and negative selection of T cells. Annu Rev Immunol (2003) 21:139–76 10.1146/annurev.immunol.21.120601.141107 12414722

[30] Juang J Ebert PJ Feng D Garcia KC Krogsgaard M Davis MM Peptide-MHC heterodimers show that thymic positive selection requires a more restricted set of self-peptides than negative selection. J Exp Med (2010) 207(6):1223–34 10.1084/jem.20092170 20457759 PMC2882826

[31] Gallegos AM Bevan MJ Central tolerance: good but imperfect. Immunol Rev (2006) 209:290–6 10.1111/j.0105-2896.2006.00348.x 16448550

[32] Ashton-Rickardt PG Studying T-cell repertoire selection using fetal thymus organ culture. Methods Mol Biol (2007) 380:171–84 10.1007/978-1-59745-395-0_10 17876093

[33] Griesemer AD Sorenson EC Hardy MA The role of the thymus in tolerance. Transplantation (2010) 90:465–74 10.1097/TP.0b013e3181e7e54f 20555306 PMC2933313

[34] Kelly K Pilarski L Shortman K Scollay R CD4+CD8+ cells are rare among in vitro activated mouse or human T lymphocytes. Cell Immunol (1988) 117:414–24 10.1016/0008-8749(88)90130-X 3264216

[35] Tikhonova AN Van Laethem F Hanada K Lu J Pobezinsky LA Hong C αβ T cell receptors that do not undergo major histocompatibility complex-specific thymic selection possess antibody-like recognition specificities. Immunity (2012) 36:79–91 10.1016/j.immuni.2011.11.013 22209676 PMC3268851

[36] Van Laethem F Tikhonova AN Pobezinsky LA Tai X Kimura MY Le Saout C Lck availability during thymic selection determines the recognition specificity of the T cell repertoire. Cell (2013) 154:1326–41 10.1016/j.cell.2013.08.009 24034254 PMC3792650

[37] Classon BJ Brown MH Garnett D Somoza C Barclay AN Willis AC The hinge region of the CD8 alpha chain: structure, antigenicity, and utility in expression of immunoglobulin superfamily domains. Int Immunol (1992) 4:215–25 10.1093/intimm/4.2.215 1377946

[38] Rudd PM Wormald MR Stanfield RL Huang M Mattsson N Speir JA Roles for glycosylation of cell surface receptors involved in cellular immune recognition. J Mol Biol (1999) 293:351–66 10.1006/jmbi.1999.3104 10529350

[39] Moody AM North SJ Reinhold B Van Dyken SJ Rogers ME Panico M Sialic acid capping of CD8beta core 1-O-glycans controls thymocyte-major histocompatibility complex class I interaction. J Biol Chem (2003) 278:7240–6 10.1074/jbc.M210468200 12459555

[40] Singer A Adoro S Park JH Lineage fate and intense debate: myths, models and mechanisms of CD4- versus CD8-lineage choice. Nat Rev Immunol (2008) 8:788–801 10.1038/nri2416 18802443 PMC2760737

[41] Itano A Robey E Highly efficient selection of CD4 and CD8 lineage thymocytes supports an instructive model of lineage commitment. Immunity (2000) 12:383–9 10.1016/S1074-7613(00)80190-9 10795736

[42] Wiest DL Yuan L Jefferson J Benveniste P Tsokos M Klausner RD Regulation of T cell receptor expression in immature CD4+CD8+ thymocytes by p56lck tyrosine kinase: basis for differential signaling by CD4 and CD8 in immature thymocytes expressing both coreceptor molecules. J Exp Med (1993) 178:1701–12 10.1084/jem.178.5.1701 8228817 PMC2191226

[43] Itano A Salmon P Kioussis D Tolaini M Corbella P Robey E The cytoplasmic domain of CD4 promotes the development of CD4 lineage T cells. J Exp Med (1996) 83:731–41 10.1084/jem.183.3.731 8642277 PMC2192343

[44] Holst J Wang H Eder KD Workman CJ Boyd KL Baquet Z Scalable signaling mediated by T cell antigen receptor-CD3 ITAMs ensures effective negative selection and prevents autoimmunity. Nat Immunol (2008) 9:658–66 10.1038/ni.1611 18469818

[45] Singer A New perspectives on a developmental dilemma: the kinetic signaling model and the importance of signal duration for the CD4/CD8 lineage decision. Curr Opin Immunol (2002) 14:207–15 10.1016/S0952-7915(02)00323-0 11869894

[46] McCaughtry TM Etzensperger R Alag A Tai X Kurtulus S Park JH Conditional deletion of cytokine receptor chains reveals that IL-7 and IL-15 specify CD8 cytotoxic lineage fate in the thymus. J Exp Med (2012) 209:2263–76 10.1084/jem.20121505 23109710 PMC3501363

[47] Adoro S Park JH Singer A Coreceptor gene “imprinting:” a genetic solution to a developmental dilemma in T cells. Cell Cycle (2012) 11:833–4 10.4161/cc.11.5.19596 22333594 PMC6690586

[48] Wang XX Li Y Yin Y Mo M Wang Q Gao W Affinity maturation of human CD4 by yeast surface display and crystal structure of a CD4-HLA-DR1 complex. Proc Natl Acad Sci U S A (2011) 108:15960–5 10.1073/pnas.1109438108 21900604 PMC3179091

[49] Mosyak L Zaller DM Wiley DC The structure of HLA-DM, the peptide exchange catalyst that loads antigen onto class II MHC molecules during antigen presentation. Immunity (1998) 9:377–83 10.1016/S1074-7613(00)80620-2 9768757

[50] Kern PS Teng M-K Smolyar A Liu J-H Liu J Hussey RE Structural basis of CD8 co-receptor function revealed by crystallographic analysis of a murine CD8αα ectodomain fragment in complex with H-2Kb. Immunity (1998) 9:519–30 10.1016/S1074-7613(00)80635-4 9806638

[51] Liu Y Xiong Y Naidenko OV Liu JH Zhang R Joachimiak A The crystal structure of a TL/CD8alphaalpha complex at 2.1 A resolution: implications for modulation of T cell activation and memory. Immunity (2003) 18:205–15 10.1016/S1074-7613(03)00027-X 12594948

[52] Salter RD Benjamin RJ Wesley PK Buxton SE Garrett TP Clayberger C A binding site for the T-cell co-receptor CD8 on the alpha 3 domain of HLA-A2. Nature (1990) 45:41–6 10.1038/345041a0 2109837

[53] Durairaj M Sharma R Varghese JC Kane KP Requirement for Q226, but not multiple charged residues, in the class I MHC CD loop/D strand for TCR-activated CD8 accessory function. Eur J Immunol (2003) 33:676–84 10.1002/eji.200323499 12616488

[54] Cole DK Pumphrey NJ Boulter JM Sami M Bell JI Gostick E Human TCR-binding affinity is governed by MHC class restriction. J Immunol (2007) 178:5727–34 17442956 10.4049/jimmunol.178.9.5727

[55] van der Merwe PA Davis SJ Molecular interactions mediating T cell antigen recognition. Annu Rev Immunol (2003) 21:659–84 10.1146/annurev.immunol.21.120601.141036 12615890

[56] Kim ST Takeuchi K Sun Z-YJ Touma M Castro CE Fahmy A The αβ T cell receptor is an anisotropic mechanosensor. J Biol Chem (2009) 284:31028–37 10.1074/jbc.M109.052712 19755427 PMC2781503

[57] Zhu C Jiang N Huang J Zarnitsyna VI Evavold BD Insights from in situ analysis of TCR-pMHC recognition: response of an interaction network. Immunol Rev (2013) 251:49–64 10.1111/imr.12016 23278740 PMC3539230

[58] Sinclair C Bains I Yates AJ Seddon B Asymmetric thymocyte death underlies the CD4:CD8 T-cell ratio in the adaptive immune system. Proc Natl Acad Sci U S A (2013) 110:E2905–14 10.1073/pnas.1304859110 23858460 PMC3732981

[59] Teh HS Kisielow P Scott B Kishi H Uematsu Y Blüthmann H Thymic major histocompatibility complex antigens and the alpha beta T-cell receptor determine the CD4/CD8 phenotype of T cells. Nature (1988) 335:229–33 10.1038/335229a0 2970593

